# Orthodontic and oral health literacy in adults

**DOI:** 10.1371/journal.pone.0273328

**Published:** 2022-08-18

**Authors:** V. Wallace McCarlie, Morgan E. Phillips, Barry D. Price, Peyton B. Taylor, George J. Eckert, Kelton T. Stewart

**Affiliations:** 1 Division of Orthodontics and Dentofacial Orthopedics, Department of Pediatric Dentistry and Orthodontics, School of Dental Medicine, East Carolina University, Greenville, North Carolina, United States of America; 2 Department of Biostatistics and Health Data Science, Indiana University School of Medicine, Indianapolis, Indiana, United States of America; 3 Department of Orthodontics and Oral Facial Genetics, Indiana University School of Dentistry, Indianapolis, Indiana, United States of America; International Medical University, MALAYSIA

## Abstract

**Objective:**

The primary aim of the study was to determine levels of literacy in both oral health and orthodontics in an adult population. The secondary study aim was to investigate differences in literacy between males and females.

**Methods:**

Participants included individuals 18 years or older seeking dental treatment at the East Carolina University (ECU) School of Dental Medicine. To determine levels of oral health literacy (OHL) and orthodontic literacy (OrthoL), validated instruments were administered, including the Rapid Estimate of Adult Literacy in Medicine and Dentistry, the Oral Health Literacy Instrument and its separate scales, and a questionnaire on orthodontic literacy. Summary statistics were computed, and statistical significance was set at 0.05.

**Results:**

One hundred seventy-two individuals participated in the study and had a mean age of 55.03 (range:18–88). Greater than 70% of the sampled population exhibited inadequate or marginal oral health knowledge. Additionally, greater than 70% of the sample possessed no more than an 8th grade reading level, with regard to basic medical and dental terms. Higher education was weakly associated with higher OrthoL and OHL. Higher age was also weakly associated with lower OrthoL and OHL. Males on average exhibited significantly higher OHL (p < .05) but there were no OrthoL differences between males and females. Dental visit frequency was not associated with OrthoL or OHL.

**Conclusion:**

Low levels of OrthoL and OHL were observed in the study. While males demonstrated a higher level of OHL than females, neither age nor the occurrence of dental appointments significantly influenced levels of literacy.

## Introduction

Both medicine and dentistry have become increasingly aware that health literacy plays or may play an important role in patient outcomes [1–3]. Oral health literacy is defined as the degree to which individuals have the capacity to obtain, process and understand basic oral health information and access services needed to make appropriate health decision [3, 4]. We conceptualize orthodontic literacy similarly, specifically related to orthodontics within this framework. A 2011 systematic review highlighted that there appears to be an inverse relationship between health literacy and utilization of health care services. For example, those with decreased health literacy showed increased hospitalizations and “emergency care” utilization [5]. Additionally, among aged groups in this referenced study, those with low health literacy exhibited “poorer overall health status and higher mortality.” In pediatric dentistry, it has been shown that children with “poorer reported oral health status” have caregivers with low literacy [6]. In adolescents, one study showed that those “with lower levels of oral health literacy had a larger number of teeth with cavitated carious lesions” [7].

Studies also show that physicians overestimate their patients’ health literacy levels and are unable to determine at-risk patients for low health literacy [8, 9]. It has also been noted that low oral health literacy (OHL) is a barrier to optimal patient care with potentially suboptimal oral health outcomes a result [10]. Thus, the concept of health literacy has emerged as an approach for enhancing health interventions [11].

Additionally, general oral health and orthodontic literacy, are highly impactful to clinical practice. Understanding where a practice’s patient pool is in terms of baseline levels of orthodontic and oral health literacy may be critical not only for patient compliance, but also in terms of providing access to care where it currently does not exist. There is evidence indicating a practitioner’s focus on increasing patient health literacy will increase patient trust in the practitioner, referrals to the practice, and augment the practice’s ability to educate patients about treatment [12]. This adult study is also relevant to clinicians since, according to the 2015 AAO Economics of Orthodontics Survey, “more than one in four orthodontic patients are now adults.”

Thus, our primary aim was to determine levels of literacy in both orthodontics and oral health in an adult population in an area that has been described as underserved [13]. The dental school, where the study participants were recruited, is situated in the 11^th^ most urban county in North Carolina and we anticipate the obtained results would be consistent with other similar places throughout the United States. The secondary study aim was to investigate differences in literacy between males and females within the sampled population, since previous research has reported differences between males and females [14].

## Materials and methods

This study was reviewed and approved by the East Carolina University (ECU) & Medical Center Institutional Review Board (IRB#14000398). The established inclusion criteria included adults (18 years or older), seeking treatment at the ECU School of Dental Medicine, who did not have a cognitive, visual, or hearing impairment. Eligible individuals needed to have at least a limited understanding of English and be able to verbally communicate. To our knowledge, no one in our study spoke English as a second language. Those excluded from the study included children and any adult that had a self-reported cognitive, visual, or hearing impairment, or who had no understanding of English. After providing written consent, participants were given a short screening form to ascertain basic demographic data. One hundred and seventy-two individuals participated in the study. They were also asked to complete multiple validated instruments, including the Oral Health Literacy Instrument (OHLI) and its separate scales (knowledge, comprehension, numeracy, total) [15], the Rapid Estimate of Adult Literacy in Medicine and Dentistry (REALMD-20) [16], and a questionnaire designed to test knowledge related to orthodontic literacy [17]. The range of scores possible in this study for each separate scale was 0 to 100 (OHLI knowledge, OHLI total, and Ortho literacy), 0 to 50 (OHLI comprehension and OHLI numeracy), or 0 to 20 (REALMD-20).

The OHLI knowledge scale (17 questions) required participants to identify an oral structure, treatment device, or home care oral hygiene product that matches a label on a picture. The comprehension scale (29 questions) required the participants to read a passage that included a missing word and fill in the blank from four options. The numeracy scale (5 prescription questions) presented various prescription labels with questions that the participant must accurately describe or calculate. An example of this would be: How many times can you refill this medication? The total OHLI scale involved a combination of these three components. The REALMD-20 is a list of 20 medical and dental terms that the participant must read and pronounce aloud. The questionnaire relating to orthodontic literacy (OrthoL) is a series of 12 basic orthodontic questions the participants respond to, either choosing a given option or filling in the blank. All OHLI scales and the REALMD-20 constituted the OHL scales for this study. The OrthoL questionnaire was the only instrument utilized to investigate patient literacy regarding orthodontics.

Descriptive statistics were computed for OHL and OrthoL scales. The association of gender with OHL and OrthoL scales was evaluated using a Kruskal-Wallis test. The associations of age, education, and dental visit frequency with the OHL and OrthoL scales were evaluated using Spearman correlation coefficients. Neither race nor ethnicity was collected from the patients and these parameters were not included in the statistical analysis. Multiple-variable linear regression analyses were used to examine the effects of the factors simultaneously on the OHL and OrthoL scales. The associations among the literacy scales were evaluated using Pearson correlation coefficients. The study was designed a priori to have at least 80% power to detect an OHLI difference of 5 between males and females, assuming at least 64 subjects in each gender, a 5% significance level, and standard deviation of 10.

## Results

Table 1 shows the demographic distribution of the study subjects. The mean age was 55 years old and 63% were female. The mean scores for the OHL and OrthoL scales are presented in Table 2. In general, the standard deviations were relatively high for the scales that measured OHLI knowledge and OrthoL, indicating more variability in the responses.

**Table 1:** Sample descriptive statistics.

Parameter		n (%)
Gender	F	108 (63%)
	M	64 (37%)
Education	Less than high school	5 (3%)
	High school	36 (21%)
	Some college	61 (36%)
	College	69 (40%)
Dental Frequency	Every 3–6 mo	63 (37%)
	Every year	39 (23%)
	Every 2–3 yr	30 (18%)
	Only when pain	35 (21%)
	Never	2 (1%)
Age	Mean (SD^a^)	55.03 (15.73)
	Minimum, Maximum	18, 88

^a^SD indicates standard deviation.

**Table 2:** Oral health and orthodontic literacy scales.

Literacy Instrument		n	Mean	SD^a^
OHLI	Knowledge	168	57.00	26.12
	Comprehension	167	41.79	8.05
	Numeracy	172	39.89	7.53
	Total	166	81.60	13.59
REALMD-20		172	16.43	3.44
Ortho Literacy		159	54.32	25.32

^a^SD indicates standard deviation.

The analyses first explored each of the demographic variables against the literacy scales. These are represented in Tables 3–6. Higher age was weakly associated with lower literacy, higher education was weakly associated with higher literacy, and dental visit frequency was not associated with literacy (Tables 4–6). Males had higher OHLI numeracy, OHLI total, and REALMD-20 than females (Table 3). Table 5 suggests some patterns by education level and the main analyses treated education level as a continuous variable. This was also true for education in the multivariable analyses (Table 7), in order to make full use of the data and provide for ordering of levels in the analysis. Both the correlation analysis (Table 4) and the multivariable analysis (Table 7) treated the dental frequency variable as a continuous variable for the same reasons.

**Table 3:** Oral health and orthodontic literacy scales by gender.

		Female		Male		
		n	Mean (SE^a^)	n	Mean (SE^a^)	p-value
OHLI	Knowledge	105	54.90 (2.66)	61	60.75 (3.10)	0.2766
	Comprehension	105	41.27 (0.84)	60	42.59 (0.91)	0.3410
	Numeracy	105	38.61 (0.71)	63	41.88 (0.95)	0.0026
	Total	104	79.77 (1.36)	60	84.64 (1.64)	0.0051
REALMD-20		105	16.08 (0.35)	63	16.92 (0.41)	0.0423
Ortho Literacy		100	54.09 (2.59)	58	54.86 (3.26)	0.9362

^a^SE indicates standard error.

**Table 4:** Oral health and orthodontic literacy scales correlated with age, education, and dental visit frequency.

		Age	p-value	Education	p-value	Dental Visit Frequency	p-value
OHLI	Knowledge	-0.31	< 0.001	0.26	< 0.001	-0.13	0.1070
	Comprehension	-0.26	< 0.001	0.24	0.0016	-0.07	0.4064
	Numeracy	-0.13	0.1012	0.32	< 0.001	-0.09	0.2650
	Total	-0.22	0.0052	0.34	< 0.001	-0.09	0.2691
REALMD-20		-0.20	0.0115	0.31	< 0.001	-0.11	0.1572
Ortho Literacy		-0.28	< 0.001	0.20	0.0135	-0.05	0.5069

**Table 5:** Oral health and orthodontic literacy scales by education level.

		Less than high school		High school		Some college		College	
		n	Mean (SE^a^)	n	Mean (SE^a^)	n	Mean (SE^a^)	n	Mean (SE^a^)
OHLI	Knowledge	4	30.88 (7.74)	34	47.40 (4.39)	60	56.37 (3.52)	68	64.01 (2.89)
	Comprehension	4	26.32 (8.44)	35	38.87 (1.79)	59	41.70 (1.01)	67	44.21 (0.45)
	Numeracy	4	28.85 (7.11)	36	36.65 (1.38)	61	39.72 (0.88)	66	42.48 (0.72)
	Total	4	55.16 (14.79)	35	75.36 (2.65)	59	81.34 (1.62)	66	86.62 (1.01)
REALMD-20		4	12.25 (2.78)	36	14.97 (0.64)	61	16.66 (0.38)	66	17.21 (0.40)
Ortho Literacy		4	29.55 (16.34)	34	46.79 (3.74)	57	56.14 (3.62)	63	58.44 (2.99)

^a^SE indicates standard error.

**Table 6:** Oral health and orthodontic literacy scales by dental visit frequency.

		Every 3–6 months		Every year		Every 2–3 years		Only when pain		Never	
		N	Mean (SE^a^)	n	Mean (SE^a^)	n	Mean (SE^a^)	n	Mean (SE^a^)	n	Mean (SE^a^)
OHLI	Knowledge	62	59.87 (3.39)	37	61.53 (3.80)	28	54.62 (4.25)	35	50.92 (5.10)	2	61.76 (26.47)
	Comprehension	63	42.17 (0.91)	37	42.92 (1.04)	28	41.73 (1.48)	33	39.35 (1.95)	2	46.71 (1.97)
	Numeracy	63	40.42 (0.90)	37	40.75 (1.15)	30	40.77 (1.22)	33	36.83 (1.58)	2	46.15 (3.85)
	Total	63	82.58 (1.60)	37	83.67 (1.93)	28	82.66 (2.17)	32	75.48 (3.17)	2	92.86 (1.87)
REALMD-20		63	16.63 (0.46)	37	16.62 (0.54)	30	16.73 (0.53)	33	15.33 (0.67)	2	18.50 (0.50)
Ortho Literacy		60	53.03 (3.51)	35	62.60 (3.92)	28	50.32 (4.47)	32	52.27 (4.53)	2	50.00 (13.64)

^a^SE indicates standard error.

**Table 7:** Multiple-variable analyses of oral health and orthodontic literacy scales as outcomes.

Outcome	Effect	Beta	SE^a^	DF^b^	t^c^	p-value
OHLI Knowledge	Intercept	70.27	12.51	158	5.62	< 0.001
	Female	-3.55	4.00	158	-0.89	0.377
	Age	-0.46	0.12	158	-3.80	< 0.001
	Education	6.65	2.36	158	2.82	0.005
	Dental Frequency	-2.76	1.61	158	-1.71	0.088
OHLI Comprehension	Intercept	36.59	3.95	157	9.27	< 0.001
	Female	-0.32	1.27	157	-0.26	0.798
	Age	-0.07	0.04	157	-1.74	0.084
	Education	3.19	0.74	157	4.30	< 0.001
	Dental Frequency	-0.47	0.51	157	-0.92	0.360
OHLI Numeracy	Intercept	35.56	3.66	159	9.71	< 0.001
	Female	-2.39	1.16	159	-2.07	0.040
	Age	-0.04	0.04	159	-0.99	0.323
	Education	2.81	0.69	159	4.10	< 0.001
	Dental Frequency	-0.46	0.47	159	-0.97	0.332
OHLI Total	Intercept	72.61	6.50	156	11.17	< 0.001
	Female	-2.73	2.07	156	-1.32	0.190
	Age	-0.11	0.06	156	-1.70	0.092
	Education	5.99	1.22	156	4.92	< 0.001
	Dental Frequency	-1.06	0.84	156	-1.26	0.211
REALMD-20	Intercept	15.30	1.75	159	8.74	< 0.001
	Female	-0.50	0.55	159	-0.90	0.368
	Age	-0.03	0.02	159	-1.52	0.130
	Education	1.04	0.33	159	3.17	0.002
	Dental Frequency	-0.19	0.23	159	-0.85	0.395
Ortho Literacy	Intercept	64.09	12.84	151	4.99	< 0.001
	Female	1.40	4.11	151	0.34	0.734
	Age	-0.43	0.13	151	-3.36	0.001
	Education	5.25	2.39	151	2.20	0.029
	Dental Frequency	-1.32	1.66	151	-0.79	0.429

^a^SE indicates standard error.

^b^DF indicates degrees of freedom.

^c^t indicates *t*-statistic.

Tables 3 and 4 present the key statistical comparisons for each demographic variable against the literacy scales. The findings suggest that dental visits are not likely learning experiences for this population and that patients are generally not becoming more oral health literate, or orthodontically literate, with regular visits to the dentist. Though generally low, we also found that males had higher OHL than females (Table 3). However, in terms of OrthoL, males and females did not exhibit significant differences (p = .936) and both demonstrated deficiency in OHL.

As expected, high correlations were observed for OHLI comprehension and OHLI numeracy with OHLI total. Moderate correlations were observed among the other OHL and OrthoL scales.

Table 7 depicts the outcomes of the multiple-variable analyses (Table 7), with the significance of each factor being evaluated, while accounting for the other factors. Being female was associated with significantly lower OHLI numeracy (p = 0.040). Older age was significantly, and negatively, associated with OHLI knowledge and Ortho Literacy (p<0.001 and p = 0.001, respectively). Higher education was significantly, and positively, associated with OHLI knowledge (p = 0.005), OHLI comprehension (p<0.001), OHLI numeracy (p<0.001), Ortho Literacy (p = 0.029), OHLI total (p<0.001), and REALMD-20 (p = 0.002). Dental visit frequency was not significantly associated with any of the OHL or OrthoL scales (p>0.05) after accounting for gender, age, and education.

## Discussion

One of the most critical findings of this study was that regular adult dental visits were not related to higher levels of either OHL or OrthoL (Tables 4, 6 and 7), which is generally consistent with findings from a recent study in adolescents where researchers found that levels of oral health literacy were independent from the “history of visiting a dentist” [7]. This strongly suggests that patients are not having “learning experiences” when they visit the dental office, similar to a situation where hospital patients “often are unable to recall their discharge diagnoses or treatment plan or to articulate how they are to take their prescribed medication” [18]. Coleman et al. indicated these patients do “not comprehend their instructions in the first place, and this may be explained, in part, by unrecognized low health literacy.”

The fact that the majority of this sample exhibited either inadequate or marginal oral health knowledge and no more than an 8^th^ grade reading level with regard to basic medical and dental terms provides evidence of significant barriers to optimal OrthoL and OHL. This is consistent with research evaluating “patient and parent understanding of the child’s orthodontic treatment in a dental school population,” where it was determined that “the vocabulary levels of the children and their parents were low; parents’ vocabulary and educational levels were correlated with their comprehension” [19].

Our study showed males and females differed significantly on two distinct measures of OHL (OHLI and REALMD-20). In contrast, there were no differences between males and females regarding OrthoL. These mixed results are consistent with the contrasting results found in other research investigating the relationship between health literacy and gender. In a review conducted by Aldin et al. it was observed that some studies have indicated that “women typically have slightly better health literacy than men,” while others do not find “significant differences between the genders” [20]. Conversely, Lee and Son found that, under particular conditions, older women with heart failure had higher cognitive impairment (15%) and inadequate health literacy (56.7%) compared to men [21]. Likewise, Waldrop-Valverde et al. completed work which showed that females scored lower on a medication management test, which the authors attributed to low numeracy [14]. Still, these relationships remain unclear and no general consensus can be made based on the current body of available literature.

Specialists may not be surprised that OrthoL appeared to be substantially low in this sample. Previous research indicated that participants poorly answered questions about risks for decay, oral hygiene, and retention [17]. Likewise, the recruited participants also poorly understood questions related to duration of treatment, frequency of visits, and types of appliances. In the previous study by Thomson et al., only 3 OrthoL questions were incorrectly answered by up to 60% of the sample. Whereas, in the current study, 7 OrthoL questions were incorrectly answered by up to 60%.

Furthermore, those involved in the current study did not understand the need to continue seeing a general dentist during orthodontic therapy. Likewise, participants did not exhibit understanding as to the consequences of not brushing well and maintaining good oral hygiene habits during orthodontic therapy. For example, there was no concept that negative consequences such as developing white spot lesions [22] or developing periodontal disease [23, 24] were the possible effects of poor oral hygiene habits during orthodontic treatment.

Moreover, the sample did not exhibit a knowledge of typical treatment time, which is consistent with recent studies within a University clinical setting in Lagos, Nigeria, examining patient and parent expectations [25], and one in Rochester, United States [26]. Nine out of ten participants did not show understanding as to the impact traditional braces might have on day-to-day living such as in playing instruments [27], speech [28, 29] and sports [30, 31]. This is generally consistent with distinct but related findings of “the impact of wearing fixed orthodontic appliances on life quality,” or “day-to-day living,” where researchers found that day-to-day living was impacted though not to the extent expected [32].

Orthodontic literacy as conceptualized in this study is the ability to obtain and understand orthodontic information in order to make reasonable or informed orthodontic care decisions. Why should this matter to orthodontists? First, improving patient outcomes or improving patients’ understanding of orthodontic conditions through increased orthodontic literacy empowers and provides patients with a higher standard of care. Second, it is likely that those who spend time to increase literacy are more trusted and receive more referrals than those who do not. For instance, a doctor that designates someone on his or her team to educate patients in order to increase their OHL or OrthoL, over a one-year period, is capable of increasing patients’ trust by 2.5 fold [12]. Additionally, Thom et al. demonstrated within a primary care model that when a person in the practice focuses on the aforementioned efforts consistently, patients are four times more likely to highly recommend their primary care provider.

The development of educational programs (both outreach, curricular and systematic instruments) focusing on these findings, will play a critical role in addressing patient oral health and orthodontic illiteracy. We expect that programs will be developed that create a foundation for protocols within the dental and specialty curriculum, providing a clearer path for patients to become more literate in orthodontics and oral health. We also anticipate that these findings and eventual protocols will be highly relevant to practitioners.

The authors acknowledge that the current study contained a few limitations. First, the conducted study only included data from a single site. However, we anticipate the obtained data would be consistent with other similar samples throughout the United States. Additionally, we did not collect information on the participants’ race or ethnicity. Based on the literature, it is possible that some of our results could be different when compared to individuals with different backgrounds. Additionally, there were several participants with missing responses for some literacy scales. Participants with missing responses were excluded from analyses of that scale. However, this was unlikely to affect the conclusions of the study because the lowest completion rate was 92% for OrthoL and 97% or higher for all other scales. We also noted that the power analysis (sample size calculation) was based on a standard deviation of 10 but some of the measurements had standard deviations that exceeded this threshold. However, we recruited nearly three times the number of required subjects, so we are confident that this did not negatively impact the study findings.

Further studies are needed to assess how OHL and OrthoL relate to orthodontic status and oral health outcomes. Additionally, similar studies that investigate the impact of race and ethnicity on OrthoL are warranted.

## Conclusions

An important finding of this study is that regular dental visits do not lead to higher oral health literacy or orthodontic literacy. Additionally, female oral health literacy was found to be significantly lower than males, specifically in the area of numeracy. Both male and female orthodontic literacy is also substantially inadequate and orthodontic literacy is generally deficient in this sample. Finally, orthodontic literacy was lower than a measure of total oral health literacy.

## Supporting information

S1 Dataset (XLSX)

## References

[1] Schiavo JH. Oral Health Literacy in the Dental Office: The Unrecognized Patient Risk Factor. Journal of Dental Hygiene. 2011;85(4):248–55. 22309865

[2] Kutner M, Greenberg E, Jin Y, Paulsen C. The Health Literacy of America’s Adults: Results From the 2003 National Assessment of Adult Literacy (NCES 2006–483). [Internet]. Washington, DC: U.S. Department of Education. National Center for Education Statistics.; 2006. Available from: http://nces.ed.gov/pubs2006/2006483.pdf

[3] Health Literacy: A Prescription to End Confusion. [Internet]. Washington, DC: Institute of Medicine: National Academies Press; 2004. Available from: https://hospitals.unm.edu/health_literacy/pdfs/HealthLiteracyExecutiveSummary.pdf 25009856

[4] People Healthy 2010 Final Review. Chapter 11: Health Communication [Internet]. Hyattsville, MD.: National Center for Health Statistics; 2012. Available from: http://www.cdc.gov/nchs/data/hpdata2010/hp2010_final_review.pdf

[5] Berkman ND, Sheridan SL, Donahue KE, Halpern DJ, Crotty K. Low Health Literacy and Health Outcomes: An Updated Systematic Review. Annals of Internal Medicine [Internet]. 2011;155(2):97–107. Available from: doi: 10.7326/0003-4819-155-2-201107190-00005 21768583

[6] Vann WF, Lee JY, Baker D, Divaris K. Oral Health Literacy among Female Caregivers: Impact on Oral Health Outcomes in Early Childhood. J Dent Res [Internet]. 2010;89(12):1395–400. Available from: doi: 10.1177/0022034510379601 20924067 PMC3123718

[7] da Costa Dutra L, de Lima LCM, Neves ÉTB, Gomes MC, de Araújo LJS, Forte FDS, et al. Adolescents with worse levels of oral health literacy have more cavitated carious lesions. PLoS ONE. 2019 Nov 1;14(11). 10.1371/journal.pone.0225176 PMC6880994 31774850

[8] Kelly PA, Haidet P. Physician overestimation of patient literacy: A potential source of health care disparities. Patient education and counseling [Internet]. 2007;66(1):119–22. Available from: http://www.sciencedirect.com/science/article/pii/S0738399106003466 doi: 10.1016/j.pec.2006.10.007 17140758

[9] Bass PF, Wilson JF, Griffith CH, Barnett DR. Residents’ Ability to Identify Patients with Poor Literacy Skills. Academic Medicine. 2002;77(10):1039–41. doi: 10.1097/00001888-200210000-00021 12377684

[10] The Invisible Barrier: Literacy and Its Relationship with Oral Health. Journal of public health dentistry. 2005;65(3):174–82. doi: 10.1111/j.1752-7325.2005.tb02808.x 16171263

[11] Sørensen K, van den Broucke S, Fullam J, Doyle G, Pelikan J, Slonska Z, et al. Health literacy and public health: A systematic review and integration of definitions and models. BMC Public Health. 2012;12(1). doi: 10.1186/1471-2458-12-80 22276600 PMC3292515

[12] Thom DH, Hessler D, Willard-Grace R, Bodenheimer T, Najmabadi A, Araujo C, et al. Does health coaching change patients’ trust in their primary care provider? Patient Education and Counseling [Internet]. 2014;96(1):135–8. Available from: http://www.sciencedirect.com/science/article/pii/S0738399114001347 doi: 10.1016/j.pec.2014.03.018 24776175

[13] Bureau of the Census. Persons in poverty. United States Census Bureau. 2000:0.

[14] Waldrop-Valverde D, Jones DL, Jayaweera D, Gonzalez P, Romero J, Ownby RL. Gender differences in medication management capacity in HIV infection: The role of health literacy and numeracy. AIDS and Behavior. 2009;13(1):46–52. doi: 10.1007/s10461-008-9425-x 18618237 PMC2606913

[15] Sabbahi DA, Lawrence HP, Limeback H, Rootman I. Development and evaluation of an oral health literacy instrument for adults. Community dentistry and oral epidemiology [Internet]. 2009;37(5):451–62. Available from: doi: 10.1111/j.1600-0528.2009.00490.x 19740249

[16] Gironda M, Der-Martirosian C, Messadi D, Holtzman J, Atchison K. A brief 20-item dental/medical health literacy screen (REALMD-20). Journal of public health dentistry [Internet]. 2013;73(1):50–5. Available from: doi: 10.1111/jphd.12005 23293880 PMC3661187

[17] Thomson AM, Cunningham SJ, Hunt NP. A comparison of information retention at an initial orthodontic consultation. The European Journal of Orthodontics. 2001;23(2):169–78. doi: 10.1093/ejo/23.2.169 11398554

[18] Coleman EA, Chugh A, Williams M V., Grigsby J, Glasheen JJ, McKenzie M, et al. Understanding and Execution of Discharge Instructions. American Journal of Medical Quality. 2013;28(5):383–91. doi: 10.1177/1062860612472931 23354870

[19] Baird JF, Kiyak HA. The uninformed orthodontic patient and parent: Treatment outcomes. American Journal of Orthodontics and Dentofacial Orthopedics. 2003 Aug 1;124(2):212–5. doi: 10.1016/s0889-5406(03)00400-1 12923519

[20] Aldin A, Chakraverty D, Baumeister A, Monsef I, Noyes J, Jakob T, et al. Gender differences in health literacy of migrants: a synthesis of qualitative evidence. Cochrane Database of Systematic Reviews [Internet]. 2019; Available from: www.cochranelibrary.com 10.1002/14651858.CD013302.pub2 PMC11635922 39665382

[21] Lee JK, Son Y-J. Gender Differences in the Impact of Cognitive Function on Health Literacy among Older Adults with Heart Failure. International Journal of Environmental Research and Public Health Article [Internet]. Available from: doi: 10.3390/ijerph15122711 30513761 PMC6313791

[22] Heymann GC, Grauer D. A Contemporary Review of White Spot Lesions in Orthodontics. Journal of Esthetic and Restorative Dentistry. 2013;25(2):85. doi: 10.1111/jerd.12013 23617380

[23] Boyd RL, Leggott PJ, Quinn RS, Eakle WS, Chambers D. Periodontal implications of orthodontic treatment in adults with reduced or normal periodontal tissues versus those of adolescents. American Journal of Orthodontics and Dentofacial Orthopedics. 1989 Sep 1;96(3):191–8. doi: 10.1016/0889-5406(89)90455-1 2773862

[24] Naranjo AA, Triviño ML, Jaramillo A, Betancourth M, Botero JE. Changes in the subgingival microbiota and periodontal parameters before and 3 months after bracket placement. American Journal of Orthodontics and Dentofacial Orthopedics. 2006 Sep 1;130(3):275.e17–275.e22. 10.1016/j.ajodo.2005.10.022 16979483

[25] Obilade OA, da Costa OO, Sanu OO. Patient/parent expectations of orthodontic treatment. International Orthodontics. 2017 Mar 1;15(1):82–102. doi: 10.1016/j.ortho.2016.12.005 28214278

[26] Michelogiannakis D, Gajendra S, Pathagunti SR, Sayers MS, Newton JT, Zhou Z, et al. Patients’ and parents’ expectations of orthodontic treatment in university settings. American Journal of Orthodontics and Dentofacial Orthopedics. 2021 Apr 1;159(4):443–52. doi: 10.1016/j.ajodo.2020.02.009 33568276

[27] Raney NA. The effects of orthodontic appliances on wind-instrument players. Journal of clinical orthodontics: JCO. 2006;40(6):384–7. 16804250

[28] Haydar B, Karabulut G, Ozkan S, Aksoy AU, Ciger S. Effects of retainers on the articulation of speech. American Journal of Orthodontics and Dentofacial Orthopedics. 1996 Nov 1;110(5):535–40. doi: 10.1016/s0889-5406(96)70062-8 8922513

[29] Stewart FN, John W, Kerr S, Taylor PJS. Appliance wear: the patient’s point of view. European Journal of Orthodontics [Internet]. 1997;19:377–82. Available from: https://academic.oup.com/ejo/article/19/4/377/518627 doi: 10.1093/ejo/19.4.377 9308258

[30] Salam S, Caldwell S. Mouthguards and Orthodontic Patients. Journal of Orthodontics [Internet]. 2008;35(4):270–5. Available from: doi: 10.1179/14653120722779 19074365

[31] Kvittem B, Hardie NA, Roettger M, Conry J. Incidence of orofacial injuries in high school sports. Journal of Public Health Dentistry. 1998;58(4):288–93. doi: 10.1111/j.1752-7325.1998.tb03011.x 10390711

[32] Zhang M, Mcgrath C, Hägg U. Patients’ Expectations and Experiences of Fixed Orthodontic Appliance Therapy Impact on Quality of Life. Angle Orthodontist [Internet]. 2007;77(2):318. Available from: http://meridian.allenpress.com/angle-orthodontist/article-pdf/77/2/318/1382216/0003-3219 doi: 10.2319/0003-3219(2007)077[0318:PEAEOF]2.0.CO;2 17319768

